# Triggering of eruptions at Axial Seamount, Juan de Fuca Ridge

**DOI:** 10.1038/s41598-020-67043-0

**Published:** 2020-06-23

**Authors:** Haley E. Cabaniss, Patricia M. Gregg, Scott L. Nooner, William W. Chadwick

**Affiliations:** 1 University of Illinois, Urbana-Champaign, 1401 W. Green St., Urbana, IL 61801 USA; 2 University of North Carolina at Wilmington, 601 S. College Rd., Wilmington, NC 28403 USA; 3 Oregon State University/Cooperative Institute for Marine Resources Studies, Hatfield Marine Science Center, 2115 SE OSU Dr., Newport, OR 97365 USA

**Keywords:** Natural hazards, Ocean sciences, Solid Earth sciences

## Abstract

The submarine volcano Axial Seamount has exhibited an inflation predictable eruption cycle, which allowed for the successful forecast of its 2015 eruption. However, the exact triggering mechanism of its eruptions remains ambiguous. The inflation predictable eruption pattern suggests a magma reservoir pressure threshold at which eruptions occur, and as such, an overpressure eruption triggering mechanism. However, recent models of volcano unrest suggest that eruptions are triggered when conditions of critical stress are achieved in the host rock surrounding a magma reservoir. We test hypotheses of eruption triggering using 3-dimensional finite element models which track stress evolution and mechanical failure in the host rock surrounding the Axial magma reservoir. In addition, we provide an assessment of model sensitivity to various temperature and non-temperature-dependent rheologies and external tectonic stresses. In this way, we assess the contribution of these conditions to volcanic deformation, crustal stress evolution, and eruption forecasts. We conclude that model rheology significantly impacts the predicted timing of through-going failure and eruption. Models consistently predict eruption at a reservoir pressure threshold of 12–14 MPa regardless of assumed model rheology, lending support to the interpretation that eruptions at Axial Seamount are triggered by reservoir overpressurization.

## Introduction

The ability to forecast volcanic unrest and evaluate precursory signals to assess whether a volcano is trending towards an eruption is a paramount goal in volcanology. The traditional approach for forecasting has been that of “pattern recognition,” in which known pre-eruptive conditions are assumed to manifest prior to the occurrence of an eruptive event^1,2^. Volcanic deformation, commonly attributed to pressurization of a magma reservoir at depth, is often cited as a clear indicator of volcanic unrest and is frequently used to assess eruption potential in this way^3–6^. While this approach has been used to successfully forecast the 2015 eruption at Axial Seamount^7^, countless examples of unforecast eruptions illustrate the complex nature of the inflation-eruption relationship^8,9^.

Despite this, geodesy remains one of the most widely used methods for monitoring active volcanic systems and deformation is a common constraint for models of unrest and eruption prediction. For example, analytical solutions such as those of Mogi^10^ or McTigue^11^ have been commonly used to model surface deformation. Such approaches have been used to calculate the expected surface deformation overlying an expanding magma reservoir and have frequently been used to assess pre-eruptive reservoir pressure conditions and eruption potential. However, such analytical approaches neglect critical crustal conditions such as temperature-dependence and inelastic behavior. Low-pressure, high-temperature deformation experiments reveal that Young’s modulus in particular is highly temperature dependent at brittle-ductile transition temperatures of 600–750 °C^12^. Furthermore, thermomechanical models of volcano deformation have shown that Young’s modulus greatly affects predicted surface deformation and strain accumulation^13–15^, and a recent investigation showed that rheology strongly impacts failure potential^16^.

A critical application of these considerations manifest in our understanding of volcanic eruption triggers and our approaches to address these phenomena numerically. The traditional paradigm in volcanology is that eruptions are triggered when the pressure within a magma reservoir exceeds the confining strength of the host rock surrounding it. However, recent investigations have challenged this paradigm citing the apparent open nature of many caldera systems as evidenced by observed degassing and lack of volatiles, and their inability to build such pressures^17^. Furthermore, the ductile nature of rock surrounding a hot magma reservoir is likely to buffer the system from mechanical failure. Some numerical findings suggest that eruptions are triggered when conditions of critical stress, induced by external phenomena such as tectonic stresses or large earthquakes, occur in the host rock supporting a magma reservoir^18,19^.

Axial Seamount is a rare example of a volcano that has had an accurate eruption forecast based on ground deformation alone. Given the (apparently) well-behaved nature of Axial Seamount’s eruption cycle, it provides an excellent case study to investigate the inflation-eruption relationship from a mechanical perspective and to test hypotheses of eruption triggering. Here we perform a series of numerical experiments to address the role of crustal rheology in models of volcano unrest. Specifically, we hindcast previous eruptions of Axial Seamount in 2011 and 2015 to investigate the role of crustal rheology on predictions of surface deformation, stress distribution, and mechanical failure of the host rock leading to eruption. These sensitivity tests strongly reinforce that rheology plays a critical role and should be considered in modeling surface deformation and forecasting eruptions.

## Modeling historical eruptions of axial seamount

**Figure 1:**
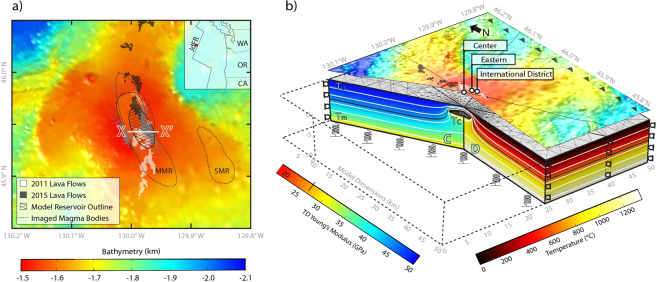
(**a**) Matlab R2018a bathymetric map showing the Axial Seamount^57^ with the location of 2011 (white)^58^ and 2015 (black)^59^ lava flows overlain (https://www.mathworks.com/products/new_products/release2018a.html). The location of earthquakes are indicated by gray dots (from Wilcock *et al*. 2016). Outlines of the main magma reservoir (MMR) and the secondary magma reservoir (SMR) identified by Arnulf *et al*.^28,29^ are shown as dashed black lines. The modeled reservoir used in this study, which was approximated from a region of high melt fraction identified in the Arnulf studies, is shown as a hatched ellipse. The line X-X’ indicates the cross-section used to generate the 2D slices in Fig. 5. (**b**) 3-D perspective view that relates the geologic setting of Axial Seamount to the COMSOL Multiphysics 5.4 FEM setup (https://www.comsol.com/release/5.4). The summit caldera along with the 2011 (white) and 2015 (black) lava flows are shown in the center of the bathymetric map overlain on the model, and primary bottom pressure instruments are identified as white circles. The model cut-away view shows the ellipsoidal magma reservoir geometry. The left-most cut plane labeled “A” shows the temperature dependent Young’s Modulus, while the right side, labeled “B” shows the host rock temperature. As in Galgana *et al*.^60^ a Winkler boundary condition is applied to the bottom of the model to account for any flexural forces, and roller boundary conditions are applied to the lateral model-bounding surfaces. Arrows on the map indicate one direction of of applied tectonic stresses, which are also applied inversely to the opposite plane (cut-away). We refer the reader to the supplement for a full discussion of the model formulation.

**Figure 2:**
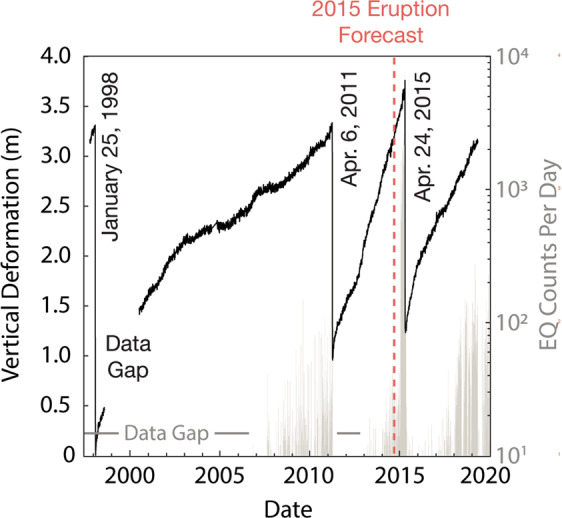
Seafloor deformation and earthquake counts at Axial Seamount provided by *in situ* seafloor instruments. Inflation and deflation record (black) is from the bottom pressure recorder at the center of the summit caldera (Center BPR—location shown in Fig. 1). Large, labeled deflation events are coincident with eruptions in 1998, 2011, and 2015, and the dashed red line indicates the time when the 2015 eruption forecast was made^25^. The light gray histogram provides earthquake (EQ) counts per day beginning in mid-2006 recorded by a combination of autonomous ocean bottom hydrophones^61^ and from ocean bottom seismometers of the Ocean Observatories Initiative’s Cabled Array^46^. Data proceeding 2006 are provided by remote monitoring networks such as the Sound Surveillance System (SOSUS) and have been widely discussed in the literature^20,44,62,63^.

Axial Seamount is a basaltic submarine volcano with a summit caldera located 480 km off the coast of Oregon at the intersection of the Juan de Fuca Ridge and the Cobb Hotspot (Fig. 1). This geologically unique setting provides Axial’s magmatic system with an abundant supply of melt, and the volcano erupts on decadal timescales^20^. In the 1990s Axial became a site for intense scientific study of volcanic and hydrothermal processes and several key time-series studies addressing surface deformation, seismicity, vent chemistry, temperature, and biology began at this time^21–24^. Since the first measurements began, seafloor deformation coincident with three eruptions was recorded in 1998, 2011, and 2015^7^ (Fig. 2). Following the 2011 eruption, researchers postulated that Axial’s eruptions are inflation-predictable, which was strongly supported by their successful forecast of the subsequent eruption in 2015^7,25–27^.

We utilize this twenty-two-year record of deformation to constrain 3D finite element models which hindcast the 2011 and 2015 eruptions of Axial Seamount. In particular, we expand upon the approach of Cabaniss *et al*.^18^ and use the COMSOL Multiphysics 5.4 modeling software to calculate stress and strain in the modeled host rock surrounding the expanding Axial magma reservoir, which is modeled as a pressurized void filled with an idealized fluid. The geometry of the simulated reservoir is approximated from a previously identified region of high melt fraction within the body of the main magma reservoir (MMR) at Axial Seamount^28,29^. We implement an ellipsoidal reservoir measuring 6 km length by 3 km width and 1 km thick to simulate this high melt region. The simulated reservoir is consistent with the location of the imaged body in relation to the summit caldera (Fig. 1) and is embedded at a depth of 1.6 km to the center, consistent with the estimated depth to the top of the magma reservoir at Axial Seamount^28,29^. A pressure boundary condition is assumed along the interior of the reservoir void space from which volumetric change and flux rate required to reproduce the observed deformation at the seafloor are estimated. As in Cabaniss *et al*.^18^, Mohr-Coulomb and tensile failure are calculated throughout the model space along with Andersonian stress orientations^30^ to determine fault type and orientation in regions where failure is calculated. For this study, eruption is defined as the first occurrence of tensile failure at the magma reservoir boundary coincident with through-going Mohr-Coulomb failure (effectively linking the reservoir to the surface).

**Figure 3:**
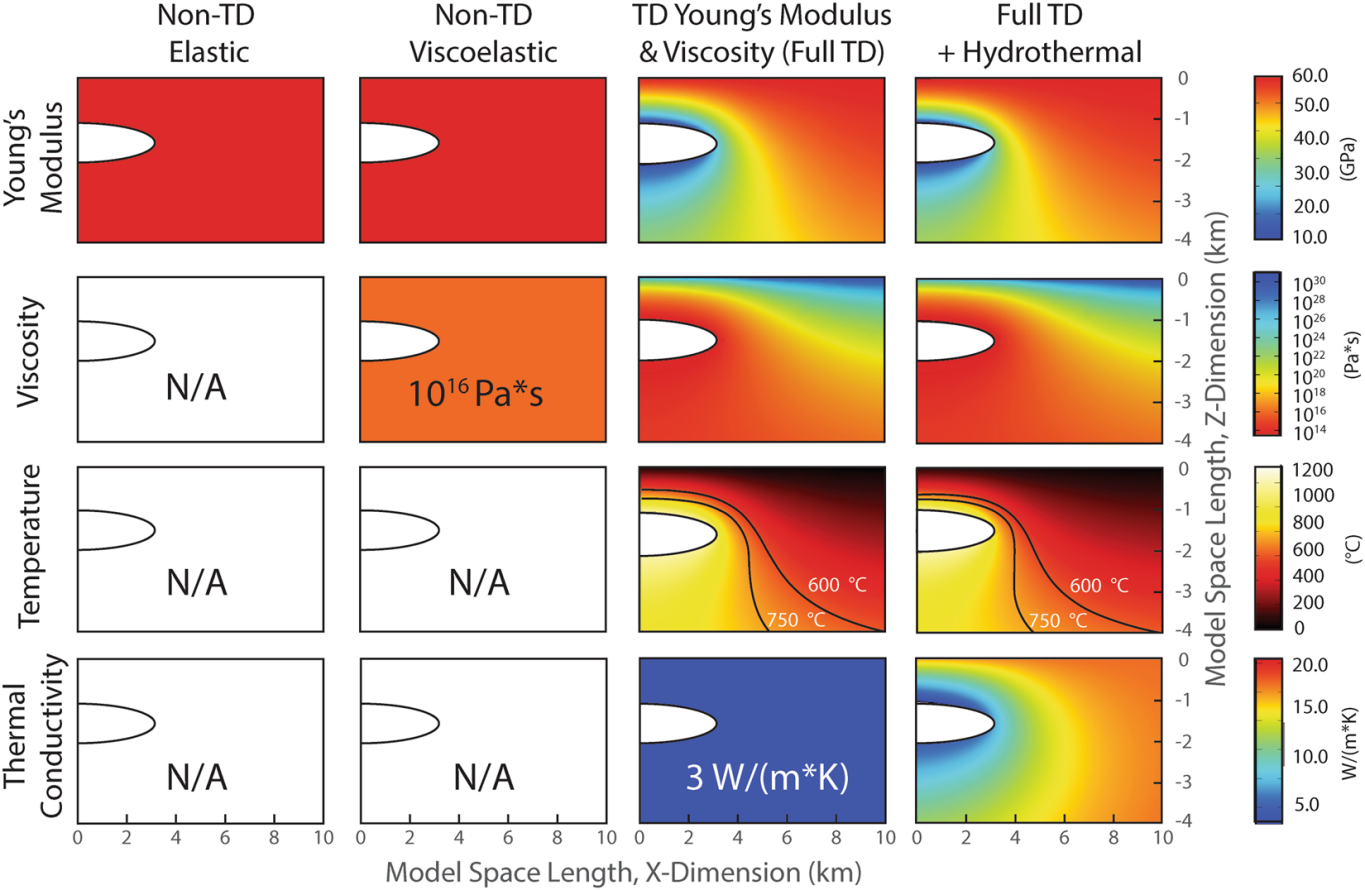
2D slices through the 3D model space show the implemented Young’s modulus, viscosity structure, thermal gradient, and thermal conductivity for each tested rheology: Non-TD Elastic (no temperature dependence); Non-TD Viscoelastic; Full TD, a viscoelastic implementation that includes a temperature-dependent Young’s Modulus and temperature-dependent viscosity; and Full TD + Hydrothermal, which incorporates increased thermal conductivity in the brittle portions of the model space. Magma reservoir is shown as the white half ellipsoid. To view the complete model-space, refer to Supplemental Fig. S1.

We perform a series of numerical experiments to investigate the effect of rheology and assumed model boundary conditions on model-predicted surface deformation, stress, and mechanical failure of the host rock supporting the Axial magma reservoir. We investigate models under four different rheologic conditions: (1) a non-temperature dependent elastic rheology, (2) a non-temperature dependent viscoelastic rheology, (3) a temperature-dependent viscoelastic rheology, and (4) a temperature-dependent viscoelastic rheology that incorporates hydrothermal circulation (Fig. 3). Additionally, the impact of local tectonic stress on Axial’s eruption cycle and magma system is investigated by applying the local tectonic forces produced by the spreading of the Juan de Fuca Ridge in an additional set of numerical experiments. In each simulation, we evaluate the stress distribution and mechanical failure of the host rock to identify plausible eruption triggers and the impact of rheology on such findings.

## Model sensitivity to rheology

**Figure 4:**
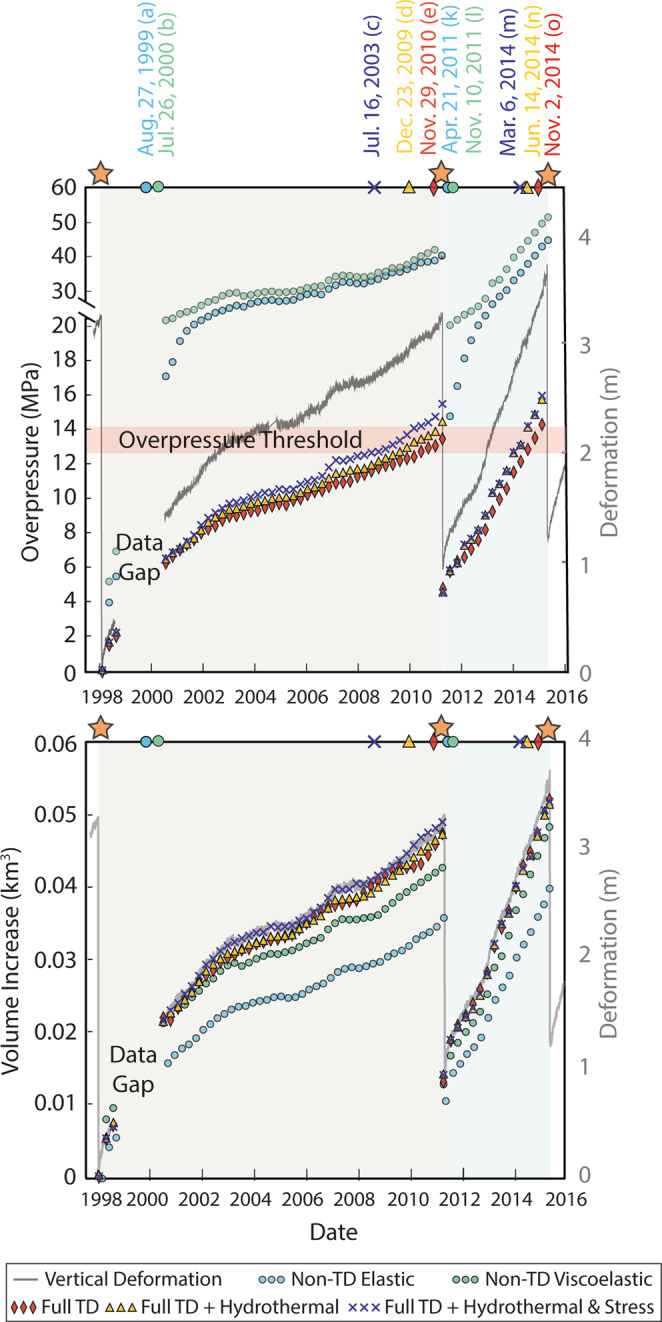
(**a**) Plot of model-predicted reservoir overpressure and (**b**) reservoir volume increase (various symbols – see legend – scale at left) necessary to reproduce the observed deformation preceding eruptions of Axial Seamount (grey line, scale at right). For clarity, shaded gray regions distinguish the 1998–2011 eruption cycle from the 2011–2015 eruption cycle in light blue. Dates above the figure indicate the time at which model-predicted eruption occurred, and stars indicate dates of observed eruptions. Note in (**a**) that the three temperature dependent models predict eruption onset at a similar overpressure threshold of 12–14 MPa (pink band).

The deformation record from Axial Seamount is used to constrain models of unrest—for each model formulation, the reservoir is pressurized such that it reproduces the observed deformation at the center of the caldera. These pressure conditions along with associated changes in reservoir volume are different for each model configuration and are shown in Fig. 4. The effect of temperature on model predictions of surface deformation is apparent. Models with a non-temperature-dependent elastic rheology require far greater reservoir overpressures to reproduce the observed surface deformation than those that incorporate a temperature-dependent rheology. Conversely, these models require *lower* reservoir volumetric change than do the temperature-dependent models. In particular, this phenomenon is revealed by comparison of the non-TD viscoelastic model to the TD viscoelastic model in Fig. 4, which otherwise have identical boundary conditions. The temperature effect is primarily due to the temperature-dependent nature of the Young’s modulus. Crustal strength is inversely proportional to temperature, and as such, the reservoir-supporting host rock in models which incorporate a temperature-dependent Young’s modulus is weaker than in those which do not. Heat promotes ductility and expansion of the weaker host rock, and as such the reservoir is able to expand longer in response to low overpressures without mechanical failure (eruption) of the host rock. For this reason, we also observe that mechanical failure occurs significantly later for temperature-dependent models than for the non-temperature-dependent models.

**Figure 5:**
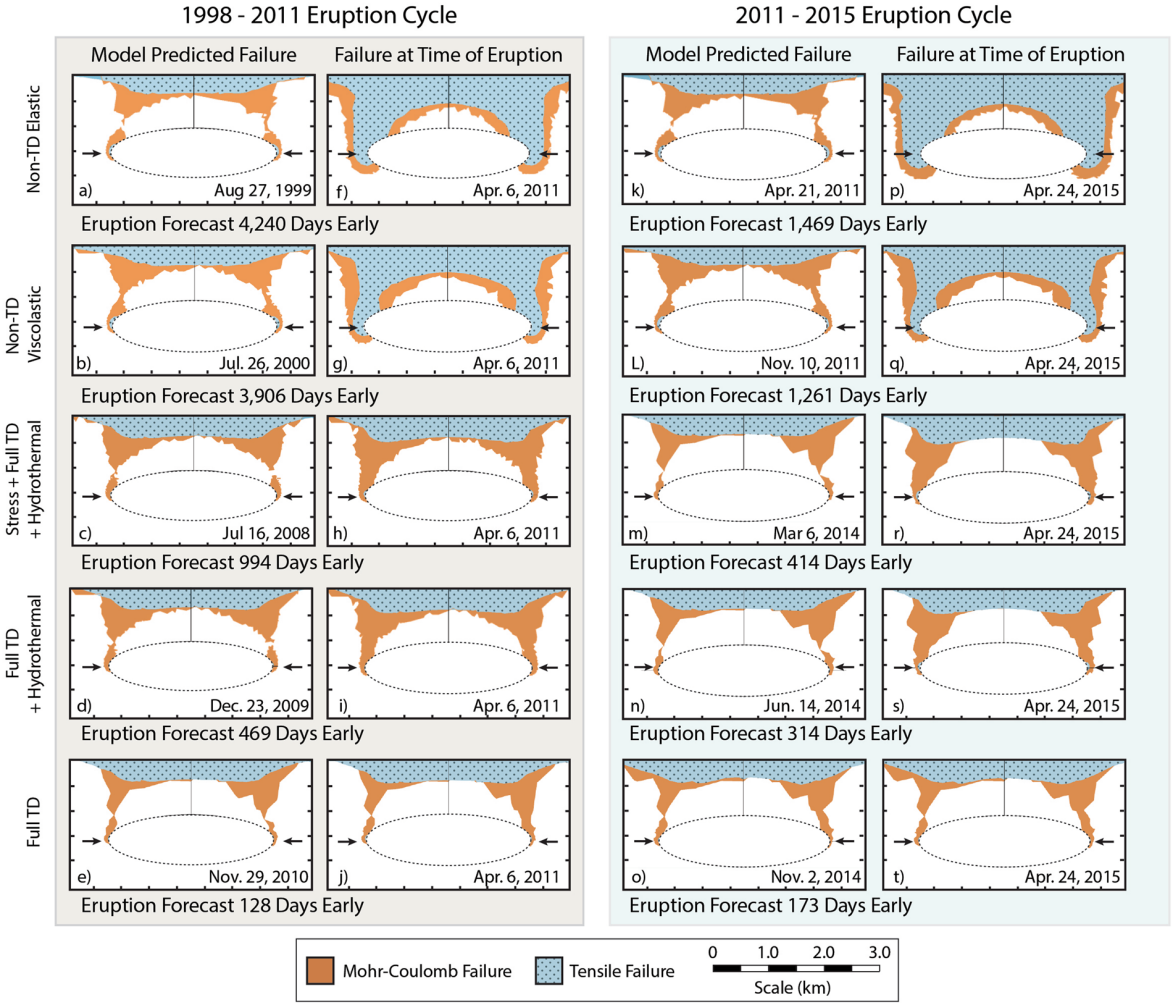
Plots of the location of Mohr-Coulomb (orange) and tensile failure (blue) shown on 2D slices through the 3D model space. The cross section from which these D slices were obtained is illustrated by the line in Fig. 1 labeled A-A’. The 1998–2011 eruption cycle is shown at left (a-j; grey background), and the 2011–2015 eruption cycle is shown at right (k-t; blue background). Failure at the time of the model predicted eruption is shown in sub-figures on the left side of each set (a-e and k-o), the time at which there is tensile failure at the reservoir boundary and through-going Mohr-Coulomb failure. The failure at the time of the observed eruption is shown in subfigures on the right of each set (f-j and p-t). Note that a single model element in tensile failure at the reservoir boundary is considered to be in tensile failure, which is difficult to illustrate in a 2D model slice, though tensile failure at the reservoir boundary occurs at each predicted eruption. Arrows point to the area of initiation for tensile failure.

We define a model as “eruptible” at the first occurrence of tensile failure along the reservoir boundary. However, we define “eruption” as the time at which through-going Mohr-Coulomb failure has occurred between the reservoir and the surface, which is always coincident with tensile failure at the reservoir boundary. Figure 5 shows the location of Mohr-Coulomb and tensile failure as a 2D slice through the 3D model space at the time of model-predicted eruption and at the time of observed eruption for each model rheology. A simple comparison of the predicted and observed eruption times shows a measure of each model’s reliability. Inflation of the magma reservoir generates widespread Mohr-Coulomb failure more rapidly for non-temperature-dependent rheologies than for those which are temperature-dependent. This phenomenon is also illuminated by the predicted vs observed eruption dates. In the most extreme example of this, non-temperature-dependent elastic models predicted the 2011 eruption should have happened more than 11 years earlier than it occurred (4,240 days) while the fully temperature-dependent models predict eruption just 128 days early. Similarly, for the 2015 eruption non-temperature-dependent models predict eruption ~4 years earlier (1,464 days) than it occurred, and the fully temperature-dependent model predicts eruption just 173 days early (Fig. 5).

Model rheologies with temperature-dependent mechanical properties (e.g. TD viscoelastic & TD viscoelastic with hydrothermal circulation in Figs. 3–5) reproduce surface deformation at similar values of reservoir overpressurization and volumetric change (Fig. 4). They also predict eruptions at dates which are closer to the observed eruption dates than the non-temperature dependent models. However, models which incorporate hydrothermal circulation require slightly higher reservoir pressurization values to reproduce the observed magnitude of deformation than TD viscoelastic models which *do not* incorporate hydrothermal circulation and they predict eruption earlier. This is because hydrothermal circulation cools the hot crust within 6 km of the surface, including the region surrounding the shallow magma reservoir centered at 1.6 km depth. The shallow crustal temperature reduction causes weakening via a decrease in the temperature-dependent Young’s Modulus. In this way, introducing hydrothermal circulation into the models causes a slightly more elastic host rock response, removing in part the thermal-buffering effect and allowing an earlier rupture of the reservoir. Cooling the shallow crustal rock via hydrothermal circulation also causes the location of the brittle-ductile transition to move closer to the magma reservoir, allowing for brittle behavior in closer proximity to the reservoir (Fig. S2).

Vigorous hydrothermal circulation is observed at three localized high-temperature vent fields in the summit caldera of Axial Seamount^20,22^. Consequently, it is expected that the models that incorporate hydrothermal circulation should yield the best results when compared to the real Axial Seamount system (more similar eruption dates). However, the models which incorporate hydrothermal circulation erupt in advance of those which do not— and far earlier than the observed eruption dates. Given the isolated nature of high temperature venting at Axial Seamount, we postulate that simulated hydrothermal circulation under the isotropic conditions assumed in the model space might not adequately represent the natural system. Instead, localized circulation may concentrate cooling and elastic behavior to focused regions in the model space, rather than impacting the entire region. However, we also acknowledge that poorly constrained factors such as the reservoir geometry (melt volume and dimensions), degree of convection, and/or the thermal regime may affect these model predictions. Results from additional numerical experiments addressing the impact of reservoir geometry on model predictions are presented in the supplementary materials. For further discussion of the impact of elastic and viscoelastic properties on results of unrest models we refer the reader to Zhan *et al*.^16^ and Head *et al*.^31^, respectively.

### Model implications for triggering of eruptions at axial seamount

The results presented here show that model rheology plays an important role in predictions of surface deformation, stress distribution, and failure. The numerical investigations also provide insight into possible mechanisms for triggering eruptions at Axial Seamount. In particular, we consider potential eruption triggers of tectonic stresses, seismicity, and critical overpressurization.

### Tectonic controls on eruption mechanics

Cabaniss *et al*.^18^ illustrated that for large, long-lived silicic caldera systems local tectonic stresses can destabilize the crust and promote eruption with relatively low magmatic flux rates over short timescales. While this effect appears to be enhanced at smaller caldera systems, the investigation did not include systems smaller than 100 km^3^ such as the Axial Seamount. Given Axial’s geologic location at the intersection of a mid-ocean ridge and a hot spot, tectonic effects may have an influence on the eruption cycle. We simulate the tectonic stress induced by plate divergence at the Juan de Fuca Ridge by imposing a spreading rate of 60 mm/year, full rate, orthogonally to the ridge axis on the surface of the model (Fig. 1). Results show that models which incorporate tectonic stresses require slightly higher reservoir overpressures and/or volume changes to reproduce the observed deformation and that these models also predict eruption earlier than observed (Figs. 4 and 5). This finding suggests that some of the stress induced by the expansion of the Axial magma reservoir is accommodated locally by extensional far field stress, allowing for more reservoir expansion with less surface deformation. However, far field extension ultimately weakens the crust and promotes eruption earlier than would be observed without any extension.

The interaction of magmatic and tectonic processes at mid-ocean ridges has been widely discussed in the literature^32–34^ but decoupling the contributions of tectonic stress in the eruption cycle of a ridge volcano is difficult^35^. Our modeling suggests that the Axial system is likely more influenced by magmatic fluxing than by tectonic stress contributions, because the latter has little effect on model predictions at the timescale of Axial’s eruption cycle. If this were *not* the case, we would expect eruptions to occur at regular intervals of time as a critical amount of cumulative tectonic extension were exceeded.

### Seismic controls on eruption mechanics

Faults are a common feature of the volcanic landscape, and seismicity, a familiar precursor to eruption^36,37^. Many volcanoes remain seismically active throughout their lifecycle, and deviation from background seismicity is thought to signal potential unrest and eruption. Faulting is expected to accommodate some reservoir overpressurization via inelastic deformation, which should prolong the pre-eruption period before co-eruption failure. Similarly, recent studies have noted instances of reduction in crustal stress and a prolonged period of unrest^38,39^. In particular, Zhan *et al*.^38^ found that high-pressure fluid injection from the Laguna del Maule magma reservoir into an adjacent fault generates periodic seismicity, which relieves stress along the magma reservoir boundary and delays eruption. Alternatively, large earthquakes are often observed moments before an eruptive event, and as such have been attributed to triggering the subsequent eruption^3,19,40–43^.

Since the late 1990s, hydrophone arrays and campaign style ocean bottom seismometer (OBS) surveys have provided almost continuous earthquake event detection at Axial Seamount^20,44^. However, hypocenters are difficult to estimate using these data, there is sparse seismic data prior to the 1998 and 2011 eruptions and observations in the years preceding the 2015 eruption are limited. In late 2014 the Ocean Observatories Initiative (OOI) Cabled Array implemented a network of seafloor sensors for real time monitoring, including a network of ocean bottom seismometers^45,46^. However, no record of seismicity yet spans the duration of an entire eruption cycle (e.g. eruption to eruption) with a consistent monitoring network. The most complete record is provided by the OOI data recorded before, during, and after the 2015 eruption^46,47^.

Seismicity provides a metric for assessing model-reliability in that predicted Mohr-Coulomb failure is anticipated in regions of observed seismicity at Axial Seamount. Our models predict top-down propagation of Mohr-Coulomb failure, whereby failure coincident with surface inflation manifests first in the near-surface regions of the model space (Fig. 5). As the reservoir continues to expand, MC failure extends downward toward the reservoir and eventually also develops at the edges of the reservoir boundary and extends upward. Shortly after, both regions of failure meet (through-going failure), signaling the model-prediction of eruption. As shown in Fig. 5, the region of through-going Mohr-Coulomb failure resembles the structure of an outward dipping fault. This model prediction is consistent with the seismic observations, which identify outward-dipping faults at Axial Seamount coincident with the locations of increased seismicity shown in Fig. 1 ^28,29,46,47^.

The record of seismicity at Axial Seamount also suggests that earthquake frequency increases quasi-exponentially in the years preceding an eruptive event^20,24,42,46^. The average moment magnitude (M_W_) for observed earthquakes in the months preceding Axial’s 2015 eruption is 0.1, with the highest magnitude earthquakes (M_W_ > 2.0) occurring during syn-eruption diking^46^. Given the magnitude of precursory seismicity, it is unlikely that the culminating eruption is triggered by a large earthquake as has been observed several times in terrestrial systems^19,48^. However, the relationship between inflation and seismicity at Axial Seamount suggests an interplay between the magmatic system and the stress state in the surrounding host rock. It is likely that inflation of the magma reservoir causes host rock stresses to build along pre-existing caldera-related faults, which generates seismicity. At some point, the cumulative stress release from seismicity should damage the host rock such that strain occurs more readily, and surface inflation occurs with less pressurization of the magma reservoir. As such, we would expect high rates of seismicity accompanying high levels of reservoir inflation to be a precursor to eruption^3^. As noted earlier our model predicted eruption dates consistently precede the observed eruption dates, forecasting eruption well in advance of the actual timing. We postulate that incorporating faults in the model space to simulate this effect might result in more accurate forecast dates. Future work should therefore address this hypothesis to investigate interdependence of magmatic and seismic processes at Axial Seamount.

### Critical overpressurization

Critical overpressurization is a commonly described mechanism for triggering eruption^1,49,50^. Under this paradigm, a stable magma reservoir is assumed to be in lithostatic equilibrium with the surrounding rock, such that the confining strength is equal to the pressure within the reservoir. As pressure builds due to injection of new melt or volatile exsolution, the system is no longer in equilibrium and the host rock responds by breaking (seismicity) and deforming. Eventually, pressure in excess of the host rock confining strength builds sufficiently to trigger eruption. As reservoir pressure is released, rapid underpressurization occurs, triggering the collapse of the overlying roof rock into resultant space in the reservoir^50^. Studies of dike propagation have revealed that pressures of 10–40 MPa are required to initiate diking, and as such, modeling studies have frequently cited 10–40 MPa of pressure as the amount required to trigger eruption in this way^51^.

However, this paradigm has been widely debated in recent years as quantitative constraints on critical overpressure to drive eruption remain uncertain. Furthermore, vent degassing and the lack of volatile elements observed in many geochemical analyses suggest the open nature of some caldera systems and their inability to build large overpressures^17^. This appears to be the case at Axial Seamount, where continuous CO_2_ flux from the hydrothermal system is observed, suggesting relatively steady-state volatile exsolution and release with no evidence for CO_2_ build up between eruptions and sudden loss during eruptions^52,53^. Therefore, volatile accumulation does not appear to be a major contributor to triggering eruptions. However, the nature of the inflation-eruption relationship observed at Axial Seamount suggests a critical deformation threshold after which eruptions are triggered, and if the surface deformation and reservoir inflation are linearly related, the observed deformation threshold may actually reflect a reservoir pressure threshold. Axial Seamount appears to have a near-continuous supply of melt as evidenced by stable inflation of the caldera floor with time. Therefore, as long as the melt supply is sufficiently high and consistent, overpressures should be able to build within the magma reservoir despite a steady loss of CO_2_. If eruptions of Axial Seamount are triggered via overpressurization, we would expect models to show a similar magnitude of reservoir pressure preceding predicted eruptions in 2011 and 2015. Conversely, dissimilar pressure conditions would suggest that eruptions are driven by external stress conditions. Nevertheless, the clear deformation threshold suggests similar pre-eruption conditions to trigger eruption. Gregg *et al*.^13^ found that flexure in the roof overlying a magma reservoir promoted top-down faulting which may ultimately trigger eruption of large basaltic caldera systems^13^. They postulated that the determining factor for this style of eruption was the roof aspect ratio (height of overlying roof rock/area of reservoir). Because rheology impacts a magma reservoir’s ability to expand and change its aspect ratio, the model sensitivity tests to rheology also provide the opportunity to assess whether eruptions of Axial Seamount may be triggered by flexure induced faulting.

**Figure 6:**
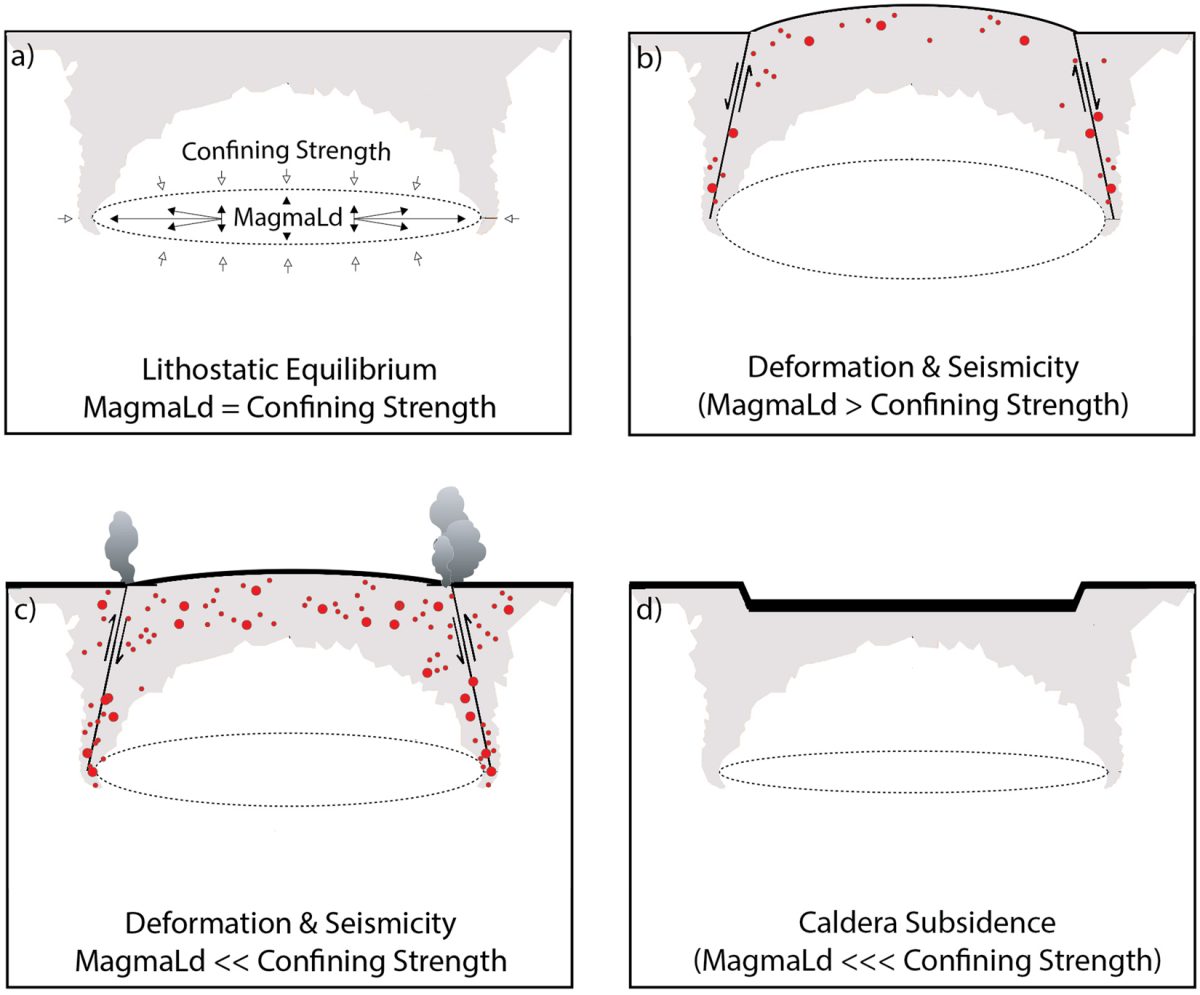
Conceptual diagram to illustrate the mechanism for triggering eruptions at Axial Seamount. Diagrams are *not* to scale, and gray shading is shown to relate model predictions of Mohr-Coulomb failure to the area of anticipated seismicity in this context. When the pressurization of the magma reservoir is equal to the confining strength of the rock surrounding it, the system is in a state of lithostatic equilibrium and seismicity and surface deformation are not observed (**a**). As the magma reservoir pressurizes (**b**) microseismicity, represented by red dots, is observed on shallow faults which accommodate some deformation of the host rock. Deformation and seismicity likely relieve some of the stresses induced on the host rock by pressurization of the reservoir and buffer the system from eruption. Eventually the pressure threshold of ~12–14 MPa is exceeded, at which time high rates of seismicity are observed as magma is intruded to the surface during an eruption (**c**). Rapid underpressurization of the magma reservoir immediately after eruption causes subsidence of the surface over magma reservoir (**d**).

Rheology sensitivity tests reveal that modeled eruptions at Axial Seamount consistently occur when the pressure boundary condition exceeds 12–14 MPa (Fig. 4a) regardless of rheological implementation. While we note that this overpressure threshold is specific for the geometry and depth of the Axial magma system (see supplement for discussion of geometry), this finding is particularly interesting given that model rheology significantly impacts the calculated volumetric change (influx). As such it seems probable that eruptions of Axial Seamount are dominantly triggered by critical overpressure stress state. Additionally, the link between inflation and seismicity suggests some stress dependence, and we postulate that fault slip accommodates some of the stresses induced by the inflation of the magma reservoir. Modeled eruption timing is proceeded by significant Mohr-Coulomb failure, which should coincide with increased seismicity such as is observed in the build-up to the 2015 eruption at Axial Seamount. Therefore, we postulate that the eruption cycle of Axial Seamount includes: (1) inflation of the magma reservoir stresses the surrounding host rock, causing increased microseismicity and deforming the rock. (2) Microseismicity relieves stress and damages the rock such that strain occurs more readily, and the deformation of the surface subsequently occurs with less reservoir pressurization. (3) Despite the stress relief through microsesimicity, eventually a pressure threshold of 12–14 MPa is exceeded in the reservoir, at which time eruption occurs (Fig. 6). This hypothesis is supported by a recent investigation which distinguished contributions from faulting and reservoir expansion to total surface deformation at Axial Seamount and found that fault slip was an important contribution to total deformation for the 2015 eruption cycle^54^.

The timing of our model predicted eruptions consistently occurs in advance of observed eruptions regardless of chosen rheology. However, the models do not simulate or take into account stress release via seismicity. Therefore, it is possible that the FEMs used in this study pre-predict eruption because they achieve the critical reservoir pressure threshold in advance of when it would occur if a condition to relieve stress through faulting were implemented. As such, future efforts should consider stress release to test the hypothesis that seismicity serves as both an eruption precursor and a buffer at the Axial Seamount.

## Conclusions

In recent years greater complexities have been revealed in the processes responsible for triggering volcanic eruptions. Increasingly, phenomena which operate externally to a volcanic system (distant seismicity, tectonic stress, etc.) are being attributed to triggering their eruptions. However, at the Axial Seamount numerical experiments indicate that eruptions are likely triggered by critical overpressurization of the magma reservoir and have little reliance on external fault or earthquake triggering.

A series of numerical experiments were performed to assess the impact of rheology on predictions of volcanic deformation, stress state, and mechanical failure of the host rock surrounding the Axial Seamount magma reservoir. These model sensitivity tests reveal that rheology strongly impacts predictions of volcanic unrest. In particular, we show that non-temperature-dependent models require an unrealistically high pressure condition to reproduce the observed deformation at Axial Seamount. Similarly, we show that eruption onset is predicted years in advance of that observed for models which do not incorporate temperature dependence. Therefore, temperature-dependence is critical to include for such modeling efforts.

Numerical results suggest that internal, magmatic processes likely drive external processes (seismicity), which in turn accommodate inflation of the magma reservoir precursory to eruption. We postulate that seismicity and fault slip on caldera-related faults work to relieve some of the host rock stresses induced by the expansion of the magma reservoir and serve as an eruption buffer rather than a catalyst (delaying the eruption onset). Ultimately, eruptions appear to be triggered by overpressurization of the magma reservoir when a critical threshold of ~12–14 MPa is exceeded.

Given the inflation-predictable eruption cycle observed at Axial Seamount, it has frequently been described as a “well-behaved” volcano, particularly when compared to terrestrial systems, which often exhibit less repeatable pre-eruption trends. Why is it that eruptions at Axial Seamount are predictable and related to magma overpressures while others are more difficult to predict? The physical setting at Axial Seamount appears to be simpler system as compared to terrestrial volcanoes, and we hypothesize that it is “well-behaved” for a number of reasons. In particular, the crustal thickness at Axial Seamount is far thinner than that of continental crust and is therefore likely to be more chemically and structurally homogeneous than continental crust. Under these conditions, melt migration to the surface should be less complicated and therefore more rapid, reducing time for interaction and melt evolution in the crust. Furthermore, Axial’s unique location at a hotspot and mid-ocean ridge spreading center supplies the shallow magma reservoir with a nearly-continuous supply of melt as opposed to the terrestrial systems which are rejuvenated rather infrequently. In addition, the system is in a tensional tectonic regime due to continuous plate spreading. These factors likely lend themselves to an inflation-predictable eruption cycle, in which reservoir pressure, surface deformation, and the timing of eruptions at Axial Seamount can be anticipated because they are closely linked.

## Supplementary information

Supplementary information.

## Data Availability

The COMSOL Multiphysics models informing this manuscript are currently available from the corresponding author on request. The bottom pressure recorder used to constrain surface deformation of the model are available via the Marine Geoscience Data System^55,56^.
