# Supplementary material for: Triggering of eruptions at Axial Seamount, Juan de Fuca Ridge
Source: Sci Rep. 2020 Jun 23;10:10219. doi: 10.1038/s41598-020-67043-0 (PMC7311472; doi:10.1038/s41598-020-67043-0)
Supplement: Supplementary file 1 — Supplementary information. [file 41598_2020_67043_MOESM1_ESM.pdf]

<u>Supporting Information for *Scientific Reports to Nature*</u>

Triggering of eruptions at Axial Seamount, Juan de Fuca Ridge

[1]Cabaniss, H.E., [1]Gregg, P.M., [2]Nooner, S.L., and [3]Chadwick, W.W.

[1]University of Illinois, Urbana-Champaign, 1401 W. Green St. Urbana, IL 61801, USA

[2]University of North Carolina at Wilmington, 601 S. College Rd. Wilmington, NC 28403, USA

[3]Oregon State University/Cooperative Institute for Marine Resources Studies, Hatfield Marine Science Center, 2030 SE Marine Science Dr., Newport, OR 97365, USA

*E-mail: cabanis2@illinois.edu

Supplemental Methods & Model Formulations

Building upon the three-dimensional approach of Cabaniss et al. (2018), a series of numerical experiments are conducted to investigate the effect of rheology on model-predicted surface deformation, stress, and mechanical failure of the host rock supporting the Axial magma reservoir. We investigate models under four different rheologic conditions: [1] a non-temperature dependent elastic rheology, [2] a non-temperature-dependent viscoelastic rheology, [3] a temperature-dependent viscoelastic rheology, and [4] a temperature-dependent viscoelastic rheology which incorporates hydrothermal circulation. The rheologies tested in this study are outlined in the subheadings below:

Non-Temperature Dependent Elastic Rheology

The elastic analytical solution of Mogi (1958) is frequently used to describe surface displacement in response to changes in the pressurization of a spherical magma reservoir at depth (Papoutsis et al., 2013; McTigue, 1987; Grosfils, 2007). Using this approach, horizontal (U_X) and vertical (U_Z) displacements are calculated as follows:

$$U_X = \frac{\Delta P a^3 x}{r^3} \frac{3K+4G}{2G(3K+G)} \qquad (1)$$

$$U_Z = \frac{\Delta P a^3 d}{r^3} \frac{3K+4G}{2G(3K+G)} \qquad (2)$$

Where *a* is the radius of the spherical source, *r* is the radial distance to the mid-point of the source, *d* is the depth to the center of the source, and *G* and *K* are the shear and bulk moduli, respectively. In this way, surface displacement is a function of only the geometric and elastic properties of the model.

Similar to the analytical solution of Mogi (1958), the simplest rheology investigated in this study is that of a purely elastic material with no temperature dependence. The 3D elastic COMSOL Multiphysics FEMs are therefore benchmarked to the approach of Mogi to insure consistency (Figure S3).

Non-Temperature Dependent Viscoelastic Rheology

A common description for the behavior of a viscoelastic material is a linear Maxwell model in which the instantaneous elastic response of a material is described by a spring with a stiffness, *G,* and the time-dependent viscous response is described by a dashpot with a coefficient of viscosity, η. The viscoelastic response of a material is further defined in the generalized Maxwell model, which takes into consideration that the host rock may relax at numerous relaxation times, which are defined as:

$$\tau_0 = \frac{\eta}{G_0 \mu_1} \qquad (3)$$

$$\tau_1 = \frac{3K+G_0}{3K+G_0\mu_0}\tau_0 \qquad (4)$$

$$\tau_2 = \frac{\tau_0}{\mu_0} \qquad (5)$$

where μ_0 and μ_1 are the fractional moduli.

Fung (1965) and Christensen (1971) demonstrated that the correspondence principle may be applied to calculate the time-dependent viscoelastic analytical solution from that of an elastic solution when either (1) The temperature field is spatially uniform but not time-dependent; (2) the temperature field is time-dependent but not spatially uniform (Del Negro et al., 2009; Christensen, 1971). We apply a spatially non-uniform, time-dependent temperature field and are therefore able to use the viscoelastic analytical solution, which has been calculated by applying the correspondence principle to the elastic Mogi analytical solution.

The viscoelastic analytical solution is derived by first taking the Laplace transform of the time-dependent variables in the elastic solution. The shear modulus, *G,* is replaced with its' Laplace transform, *μ(s),* and the change in pressure, *ΔP,* is replaced by its' Laplace transform, *ΔP/s,* where *s* is the Laplace transform variable. Del Negro et al. (2009) provide the Laplace transform of the shear modulus:

$$\tilde{u}(s) = \frac{s(\mu_0+\mu_1)G_0+\mu_0\mu_1 G_0^2/\eta}{s+mu_1 G_0/\eta}. \quad (6)$$

The Laplace transform of the viscoelastic analytical solution, *A(s)* is provided by plugging the Laplace transform variables into the second term of the elastic Mogi analytical solution:

$$\tilde{A}(s) = \frac{3K+4\tilde{\mu}(s)}{(2s\tilde{\mu}(s))(3K+4\tilde{\mu}(s))} \qquad (7)$$

The inverse of the Laplace transform, *A(s)* is given by:

$$A(t) = \frac{1}{2G_0}\left[\frac{3K+4G_0\mu_0}{\mu_0(3K+G_{0\mu})} - 3\frac{\eta G_0^2 e^{e^{-((G_0\,\mu_1(3K+G_0\mu_0))}/\eta(3K+G_0))t}}{\eta(3K+G_0\mu_0)(3K+G_0)}(1-\mu_0) - \left(\frac{1}{\mu_0}-1\right)e^{-\left(\frac{G_0\mu_0\mu_1}{\eta}\right)t}\right]. \ (8)$$

Multiplying equation 8 by the geometric first term in the Mogi equations (eqns. 1 and 2) provides the time-dependent viscoelastic solution for displacement, which simplifies to the elastic analytical solution at time=0.

We implement viscoelasticity into the FEM with the numerical approach of Del Negro et al. (2009) whose formulation uses the linear Maxwell model:

$$\frac{d\varepsilon}{dt} \propto \frac{\sigma}{\eta} + \frac{1}{G}\frac{d\sigma}{dt} \qquad (9)$$

Where ε is strain, σ is stress, and the shear modulus, G, is a function of the Young's modulus, E:

$$G = E/2(1 + \nu) \qquad (10)$$

Where ν is Poisson's ratio (Gregg et al., 2012). The generalized Maxwell model is described as j Maxwell working in parallel and is expressed discretely as:

$$\sigma = 2G\left(\mu_0\epsilon + \sum_{i=0}^{j} \mu_i\, q_i\right) \qquad (11)$$

$$\sum_{i=0}^{j} \mu_i = 1 \qquad (12)$$

Where μ_i is the fractional shear modulus and q_i is the solution to the differential equation of the strain rate, ε:

$$q_i + \frac{1}{\tau_i} q_i = \epsilon \qquad (13)$$

We test the accuracy of the COMSOL Multiphysics model by comparing calculated surface displacements to those of the elastic analytical solution from Mogi (1958), the time=0 viscoelastic analytical solution of Del Negro et al. (2009), the 2D numerical model of Gregg et al. (2012), and the 3D numerical model of Cabaniss et al. (2018). For this exercise, each model assumes the same source depth, reservoir radius, and instantaneous pressurization. As observed in Figure (S3), the models produce comparable results and are suitable for use in the study.

Temperature Dependent Viscosity & Young's Modulus (Fully TD)

Temperature is calculated numerically using the steady-state heat conduction equation:

$$\nabla \cdot (K \nabla T) = -Q \qquad (14)$$

Where k is the temperature-dependent thermal conductivity (Whittington et al., 2009), T is temperature, and Q is the crustal volumetric heat production, assumed to be zero. That temperature is then used to calculate the temperature-dependent viscosity at each point in the model space:

$$\eta_{td} = A_D exp\left(\frac{E_A}{R_g T}\right) \qquad (15)$$

Where A_D is the Dorn parameter, E_A is the activation energy, and R is the universal gas constant (see also Del Negro et al., 2009). The steady state temperature dependent viscosity is used as the

viscosity component of the viscoelastic formula (eqn. 8) which establishes the viscoelasticity of the model space.

Temperature dependent Young's modulus, E_{TD} is implemented in the model space (as in Gregg et al., 2013):

$$E_{TD} = E_D + \frac{E_B}{1+C_S * exp(A_S(1-T/T_{max})} \qquad (16)$$

where E_D is the Young's modulus in the ductile portions of the model space, E_B is the Young's modulus in the brittle portion of the model space, T is the temperature, and T_{max} is the is the magma chamber temperature. C_S and A_S are smoothing factors (Gregg et al., 2013).

As both the shear and bulk moduli are a function of the Young's modulus, we adopt a temperature dependent shear modulus:

$$G_{TD} = \frac{E_{TD}}{2(1+\nu)} \qquad (17)$$

and a temperature dependent bulk modulus:

$$K_{TD} = \frac{E_{TD}}{3(1-2\nu)} \qquad (18)$$

Temperature dependent viscoelastic relaxation times are implemented as:

$$\tau_{0td} = \frac{\eta_{td}}{G_{0TD}\mu_1} \qquad (19)$$

$$\tau_{1td} = \frac{3K_{td}+G_{td}}{3K_{td}+G_{td}\mu_0}\tau_{0td} \qquad (20)$$

$$\tau_{2td} = \frac{\tau_{0td}}{\mu_0} \qquad (21)$$

Fully Temperature Dependent with Hydrothermal Circulation

Hydrothermal circulation will change the thermal structure of the model space by reducing temperatures in the shallow crust <6 km or <600°C. In these regions, we increase the thermal conductivity by an enhancement factor, or Nusselt number (*Nu)*. We use the implementation of Gregg et al. (2009) to increase hydrothermal circulation in the model space:

$$k = k_0 + k_0{}^*(Nu - 1) * exp(A * (1 - \frac{T}{T_{max}})) * exp(A * (1 - \frac{z}{z_{max}})) \qquad (22)$$

Where k is the effective thermal conductivity, k_0 is the reference thermal conductivity, A is a smoothing factor, T_{max} is the cutoff maximum temperature (600°C), z is depth, and $zmax$ is the cutoff maximum depth (6 km).

Model Set-up

We utilize COMSOL Multiphysics 5.4 to develop a 3-dimensional finite element models (FEM) in which a magma reservoir is embedded within the appropriate aforementioned rheology (Fig. S5). A roller boundary condition is prescribed to the lateral margins of the model-bounding block to constrain horizontal motion, while a Winkler foundation is applied to the base of the model to account for flexure of the lithosphere and constrain vertical motion. Using the approach of Galgana et al. (2011), we apply a Winkler boundary condition using the COMSOL Multiphysics "spring foundation," and calculate for the stiffness of the spring constant, *s:*

$$s = \frac{\rho V g}{Z_{disp}} \qquad (23)$$

In this scenario, ρ refers to the density of the overlying block, g is gravity, V is the volume of the model-bounding block (e.g. xyz dimensions), and Z_{disp} is the amount of vertical displacement anticipated given some spring constant, k. Collectively $\rho g V$ account for the Force of the overlying model-bounding block on the Winkler foundation, and s is the resistance to that force (Asthenospheric resistance). For benchmarking purposes, we set the displacement to 1e-10 m such that little displacement occurs, and we are able to compare results of surface displacement calculated using Mogi models built with both the Winkler and elastic roller boundary conditions (Figure S3; Figure S4). Results of the benchmark are in good agreement, and we find that no observable differences in model-predicted surface displacement, Mohr-Coulomb failure or tensile failure are observed until the spring constant, s, is weakened by at least 6 orders of magnitude (Figure S3).

The FEM utilizes a pressurized-void approach, whereby pressures exceeding that of lithostatic are exerted as a boundary condition on the modeled reservoir:

$$MagmaLd = \Delta P + \rho_r gz \qquad (24)$$

Where ΔP is the change in reservoir pressure, ρ_r is the host rock density, g is the gravitational constant and z is the model depth. Though we prescribe a pressure boundary condition to the reservoir, we integrate along the reservoir boundary using an integration tool in COMSOL Multiphysics to calculate the change in surface area, which in 3-dimensional space, is expressed as a volume. In this way, we are able to measure changes in reservoir volume and calculate fluxes.

Quantifying Mechanical Failure

Each FEM calculates for the principal stresses in the model-space, which are then used to determine when and where both tensile and Mohr-Coulomb failure occur. Tensile failure at the reservoir boundary is a requirement for dike propagation and therefore, a model-prediction showing any amount of tensile failure along the reservoir boundary is said to be in an eruptible state. While tensile failure does not define the timing of eruption onset, it certainly precludes eruption. Mohr-Coulomb failure which connects the reservoir to the surface is defined as "through-going failure," and the time at which both tensile failure at the reservoir boundary and through-going failure occur defines a model-predicted eruption. A Mohr-Coulomb failure criterion has been incorporated in the FEM as:

$$\tau = C + f\sigma_n \qquad (25)$$

Where τ is the shear stress at failure, C is cohesion, f is the internal friction coefficient and σ_n is the mean stress normal to the failure plane.

Model Sensitivity Tests to Reservoir Geometry

The Axial system has been imaged seismically in great detail and the reservoir dimensions are well constrained (Arnulf et al., 2014; 2018). Two known bodies exist—the main magma reservoir (MMR), centered under the summit caldera, and the secondary magma reservoir (SMR) located to the east of the summit caldera. The primary storage system for the Axial Seamount is the MMR and we assume little interaction occurs between the two. For this reason, we do *not* consider the SMR in our investigation. The MMR measures 14 km long, 3 km wide, up to 1 km thick, and is located at a depth of 2.8 – 1.1 km below the seafloor (BSF). Within this body, is a smaller region of high melt fraction (38.6-65.8%) coincident with the optimal pressure source location of Nooner & Chadwick (2016), an area of known faulting, increased seismicity, and eruptive fissures (Wilcock 2016; Levy et al., 2018; Wilcock et al., 2018). Because most activity is focused in this region, our primary experiments reported on in the main text of this manuscript utilize an ellipsoidal geometry to approximate this region of high melt fraction, from here on referred to as the partial reservoir, rather than the full dimensions of the imaged Axial magma reservoir, from here on referred to the full reservoir.

We perform a series of numerical experiments to explore the effect of modeled geometry on predictions of unrest at Axial Seamount. In particular, we investigate three different magma reservoir geometries, informed by the aforementioned seismic surveys, to assess the impact of reservoir dimensions and depth on model predictions of unrest and eruption. Models reported on in the main text of the manuscript and used for comparison purposes here investigate an [1] ellipsoidal reservoir approximated by the region of high-melt fraction and at a depth of 1.1 km (from here on referred to as "Partial Res. BSF 1.1," where BSF refers to depth "below the seafloor"). We also investigate [2] an ellipsoidal reservoir of the same reservoir dimensions though centered at a depth of 2.6 km (Partial Res. BSF 2.8), and [3] an ellipsoidal reservoir which approximates the dimensions of the full reservoir identified at Axial Seamount and centered at a depth of 1.1 km (Full Res. BSF 1.1). Table S3 provides reservoir dimensions for

each tested geometry. Investigating this range in reservoir dimensions and depth informed by seismic observations allows us to identify a range of probable predictions for the evolving Axial Seamount system.

Figure S6 shows the required reservoir pressure conditions to reproduce observed deformation at Axial seamount for each of the modeled reservoir geometries and for each tested rheology reported in the main text. From this figure it is clear that the deep reservoir (Partial Res. 2.8) requires higher values of reservoir pressure to reproduce the observed deformation at Axial Seamount. Increasing the reservoir depth also increases the pressure threshold to trigger eruption, and as a result, eruption was not achieved for the partial reservoir geometry at a depth of 2.8 km BSF. This is because increasing reservoir depth increases the overburden (confining strength) acting on the system. As such, reducing the reservoir depth should have the opposite effect, leading to destabilization and eruption at lower values of reservoir pressurization and at an earlier date than predicted by models reported in this study.

Increasing the reservoir dimensions (and thus volume) to simulate the full MMR geometry (Full Res. 1.1) causes a decreases to the necessary overpressure to reproduce the observed deformation at Axial Seamount. However, reservoir pressure is dispersed over a larger magma reservoir in this scenario, and higher values of reservoir pressure are required to trigger failure of the full magma reservoir geometry model. As such, eruption was also not achieved for this reservoir geometry. These findings suggest the primary geometry, which was approximated from the region of high melt and reported on in the main text, is reasonable for this investigation. Likewise, they suggest that the alternative geometries tested serve as end member approximations rather than reasonable modeled predictions for the Axial Seamount system.

While we have not explicitly investigated the impact of reservoir thickness on model predictions, we would expect from the sensitivity tests that if the thickness (e.g. volume) of the reservoir is increased, lower values of reservoir pressure should be required to reproduce the observed deformation at Axial Seamount. Likewise, we would expect for an increase in the reservoir volume should delay an eruption prediction. Conversely, if we were to thin the reservoir, we would expect higher reservoir pressures to reproduce the observed deformation, and as such, a more rapid eruption. This assumption should hold true if the depth to the top of the reservoir is unchanged (e.g. reservoir thickness is accommodated at the bottom rather than the top of the reservoir). However, if the depth to the top of the reservoir were to be decreased to accommodate inflation of the magma reservoir, the associated decrease in confining strength should result in lower overpressures to reproduce the observed deformation and a reduction in the timing of eruption onset.

Each of the models reported on in these sensitivity tests to geometry implement an ellipsoidal reservoir shape. Because our work is focused primarily on identifying stress within the host rock surrounding an expanding magma reservoir rather than complex magma dynamics (complex reservoir geometries, composition, compressibility, etc…), an ellipsoidal reservoir shape provides the strongest approximation to a sequence of stacked sills, as observed at many volcanic systems, while reducing some computational expense. While the models reported in this study *do* contain a magma reservoir which has been rotated N30°W in order to align the modeled Juan de Fuca Ridge with the direction of extension in the model space, none of the reservoirs incorporate dip. Nooner & Chadwick (2016) identify the optimal pressure source to best fit the deformation at Axial Seamount at the time of the 2015 eruption as an prolate spheroid with the major axis dipping at 77º with major and minor axes of 2.2 and 0.38 km, respectively, and a depth to center of 3.81 km. Early in our investigation, we tested this pressure source geometry

and found this shape to be unstable for investigating mechanical failure of the host rock surrounding the magma reservoir, with through-going Mohr-Coulomb failure occurring with any amount of reservoir overpressure. This is because stress concentrates at the reservoir tip, where the reservoir thins toward its lateral margins. Tensile- and Mohr-Coulomb failure which initiate at the reservoir boundary always initiate in these regions. When the reservoir is inclined such that the tip is closest to the surface, through-going failure is easy to achieve, and the models fail with low reservoir overpressures. This finding provides further support to the notion that best-fitting deformation models do not necessarily show the geometry of the entire magma body. While we acknowledge that tilting the modeled magma reservoir will significantly impact predictions, we find that reservoir geometry is a more reliable approximation for calculating mechanical failure.

References

ARNULF, A.F., HARDING, A.J., KENT, G.M., CARBOTTE, S.M., CANALES, J.P., & NEDIMOVÍC, M.R. (2014). Anatomy of an active submarine volcano. Geology, 42(8), 655-658.

ARNULF, A.F., HARDING, A.J., KENT, G.M., & WILCOCK, W.S.D. (2018). Structure, seismicity, and accretionary processes at the hot spot-influenced Axial Seamount on the Juan de Fuca Ridge. Journal of Geophysical Research, 123(6), 4618-4646.

CABANISS, H.E., GREGG, P.M., & GROSFILS, E.B. (2018). The role of tectonic stress in triggering large silicic caldera eruptions. *Geophysical Research Letters, 45,* 3889-3895.

CHRISTENSEN, R.M. (1971). Theory of Viscoelasticity: An Introduction. New York NY: Academic Press.

DEL NEGRO, C., CURRENTI, G., & SCANDURA, D. (2009). Temperature-dependent viscoelastic modeling of ground deformation: application to Etna volcano during the 1993-1997 inflation period. *Physics of the Earth and Planetary Interiors, 172,* 299-309.

FUNG, Y.C. (1965). Foundations of Solid Mechanics. Prentice-Hall, Englewood Cliffs.

GALGANA, G.A., MCGOVERN, P.J., & GROSFILS, E.B. (2011). Evolution of large Venusian volcanoes: Insights from coupled models of lithospheric flexure and magma reservoir pressurization. *J. Geophys. Res*. **116**(E03009); 10.1029/2010JE003654.

GREGG, P.M., BEHN, M.D., LIN, J., AND GROVE, & T.L. (2009). Melt generation, crystallization, and extraction beneath segmented oceanic transform faults. *Journal of Geophysical Research, 114(B11102).* 10.1029/2008JB006100.

GREGG, P.M., DE SILVA, S.L., GROSFILS, E.B., & PARMIGIANI, J.P. (2012). Catastrophic caldera-forming eruptions: Thermomechanics and implications for eruption triggering and maximum caldera dimensions on Earth. *Journal of Volcanology and Geothermal Research 241-242,*1-12.

GREGG, P.M., DE SILVA, S.L., & GROSFILS, E.B. (2013). Thermomechanics of shallow magma chamber pressurization: Implications for the assessment of ground deformation data at active volcanoes. *Earth and Planetary Science Letters, 384,* 100-108.

GROSFILS, E.B. (2007). Magma reservoir failure on the terrestrial planets: Assessing the importance of gravitational loading in simple elastic models. *Journal of Volcanology and Geothermal Research, v.166,* 47-75.

LEVY, S., BOHNENSTIEHL, D.R., SPRINKLE, P., BOETTCHER, M.S., WILCOCK, W.S.D., TOLSTOY, M., & WALDHAUSER, F. (2018). Mechanics of fault reactivation before, during, and after the 2015 eruption of Axial Seamount. Geology, v. 46(5), p.447-450.

MCTIGUE, D.F. (1987). Elastic stress and deformation near a finite spherical magma body: resolution of the point source paradox. *Journal of Geophysical Research, 92,* 12931-12940.

MOGI, K. (1958). Relations of the eruptions of various volcanoes and the deformations of the ground surface around them. *Bulletin of the Earthquake and Research Institute, 36,*99-134.

NOONER, S.L., & CHADWICK, W.W., JR. (2016). Inflation-predictable behavior and co-eruption deformation at Axial Seamount. Science, 354(6318), 1399-1403.

PAPOUTSIS, I., PAPANIKOLAOU, X., FLOYD, M., JI, K.H., KONTOES, C., PARADISSIS, D., & ZACHARIS, V. (2013). Mapping inflation at Santorini volcano, Greece, using GPS and InSAR. *Geophysical Research Letters, 40,* 267-272.

WHITTINGTON, A.G., HOFMEISTER, A.M., & NABELEK, P.I. (2009). Temperature-dependent thermal diffusivity of the Earth's crust and implications for magmatism. *Nature, 458,* 319-321.

WILCOCK, W.S.D., DZIAK, R.P., TOLSTOY, M., CHADWICK JR., W.W., NOONER, S.L., BOHNENSTIEHL, D.R., CAPLAN-AUERBACH, J., WALDHAUSER, F., ARNULF, A.F., BAILLARD, C., LAU, T.-K., HAXEL, J.H., TAN, Y.J., GARCIA, C., LEVY, S., & MANN, M.E. (2018). The recent volcanic history of Axial Seamount: Geophysical insights into past eruption dynamics with an eye toward enhanced observations of future eruptions. Oceanography, 31(1), 114-123.

WILCOCK, W. S. D., M. TOLSTOY, F. WALDHAUSER, C. GARCIA, Y. J. TAN, D. R. BOHNENSTIEHL, J. CAPLAN-AUERBACH, R. P. DZIAK, A. ARNULF, AND M. E. MANN (2016). Seismic constraints on caldera dynamics from the 2015 Axial Seamount eruption, Science, 354, 1395-1399.

Supplemental Tables and Figures

Parameter	Description	Values
a	Spherical Source Radius for Elastic Solution, km	0.7
A_d	Dorn Parameter, Pa s	10^9
A_S	Smoothing Factor for E_{td}	12
C	Cohesion, Pa	10^6
C_S	Smoothing Factor for E_{td}	5
d	Depth to Spherical Source for Elastic Solution, km	4
E_A	Activation Energy, J mol^{-1}	1.2 x 10^5
E_B	Young's Modulus in Brittle Portions of Model, GPa	50
E_D	Young's Modulus in Ductile Portions of Model, GPa	25
f	Angle of Internal Friction, °	25
g	Gravity, m s^{-2}	9.81
E	Young's Modulus for Analytical Solutions, GPa	60
G	Shear Modulus for Analytical Solutions, GPa	E/(2(1+ν))
G_0	Initial Shear Modulus for Viscoelastic Solution, GPa	G
K	Bulk Modulus for Analytical Solutions, GPa	E/(3(1-2ν))
Pv	Prescribed Velocity, mm/year	-20 to 20
Q	Crustal Volumetric Heat Production, °C	0
R_g	Universal Gas Constant, J mol^{-1} K^{-1}	8.3114
T_c	Reservoir Temperature, °C	1200
k_0	Reference Thermal Conductivity, W m^{-1} °K^{-1}	3
Nu	Nusselt Number	8
A	Smoothing Coefficient for Hydrothermal Cooling Eqn.	0.75
Tmax	Maximum Temperature for Hydrothermal Cooling, °C	600
z_{max}	Maximum Depth for Hydrothermal Cooling, km	6

Table S1. Model Parameters

Variable	Description	Values
A(s)	Laplace Transform of the Viscoelastic Solution	Eq. S7
A(t)	Inverse of A(s)	Eq. S8
E_{td}	Temperature Dependent Young's Modulus, Pa	Eq. S16
G_{td}	Temperature Dependent Shear Modulus, GPa	Eq. S17
K_{td}	Temperature Dependent Bulk Modulus, GPa	Eq. S18
MagmaLd	Reservoir Boundary Load, MPa	Eq. S24
q_i	Solution to Differential Equation of Strain Rate	Eq. S13
r	Radial Distance to Source Midpoint in Elastic Soln., km	Calculated by FEM
s	Laplace Transform Variable	Calculated by FEM
T	Temperature, °C	Calculated by FEM
$\tilde{u}(s)$	Laplace Transform of Shear Modulus	Eq. S6
U_X	Horizontal Displacement for Elastic Solution, km	Eq. S1
U_Z	Vertical Displacement for Elastic Solution, km	Eq. S2
z	Depth, km	Calculated by FEM
ε	Strain	Calculated by FEM
ϵ	Differential Equation of Strain Rate	Eq. S13
η_{td}	Temperature-Dependent Viscosity, Pas	Eq. S15
σ	Stress, Pa	Calculated by FEM
σ_n	Normal Stress at Failure	Eq. S25; Calculated by FEM
σ_r	Stress in r Direction	Calculated by FEM
σ_ϕ	Stress in ϕ Direction	Calculated by FEM
σ_z	Stress in z Direction	Calculated by FEM
τ	Shear Stress at Failure	Eq. S25; Calculated by FEM
τ_0, τ_1, τ_2	Maxwell Relaxation Times for Viscoelastic Solution, s	Eq. S3, S4, S5
$\tau_{0td}, \tau_{1td}, \tau_{2td}$	Temperature Dependent Maxwell Relaxation times, s	Eq. S19, S20, S21
k	Effective Thermal Conductivity	Eq. 22
s	Spring Constant for Winkler Boundary Condition	Eq. 23

Table S2. Model Variables

	Full Res. BSF 1.1	Partial Res. BSF 1.1	Partial Res. BSF 2.8
**Depth (km)**	1.1	1.1	2.8
**Length (km)**	14	6	6
**Width (km)**	3	3	3
**Thickness (km)**	1	1	1

Table S3. Modeled Magma Reservoir Geometries

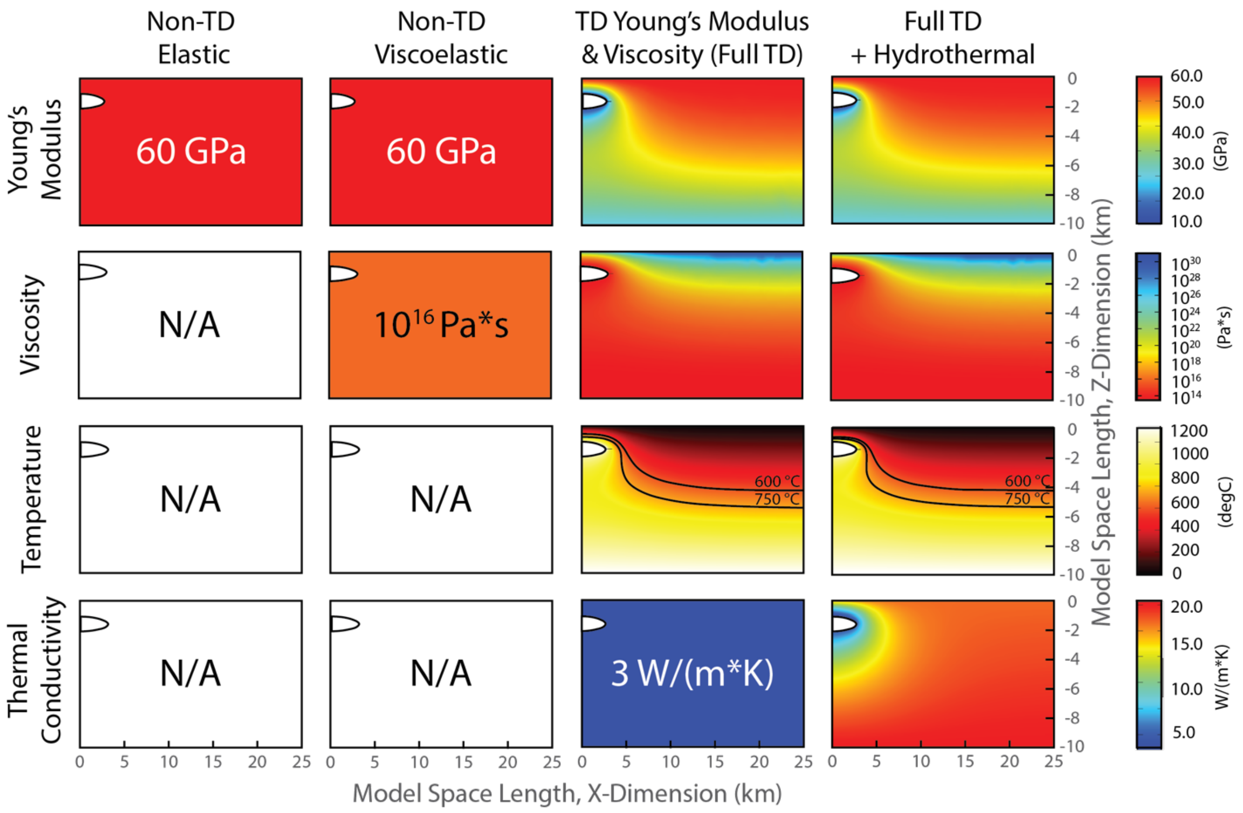

Figure S1. 2D slices through the 3D model space show the implemented Young's modulus, viscosity structure, thermal gradient, and thermal conductivity for each tested rheology: Non-TD Elastic (no temperature dependence); Non-TD Viscoelastic; Full TD, a viscoelastic implementation that includes a temperature-dependent Young's Modulus and temperature-dependent viscosity; and Full TD + Hydrothermal, which incorporates increased thermal conductivity in the brittle portions of the model space. Magma reservoir is in the upper left in each slice.

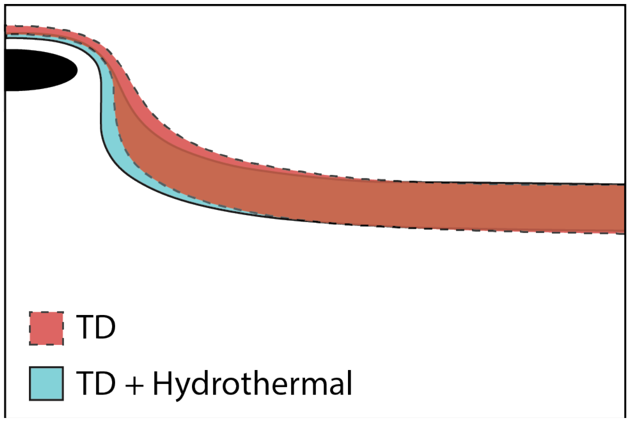

Figure S2: Illustrates the effect of hydrothermal circulation on the location of the brittle-ductile transition in the model space.

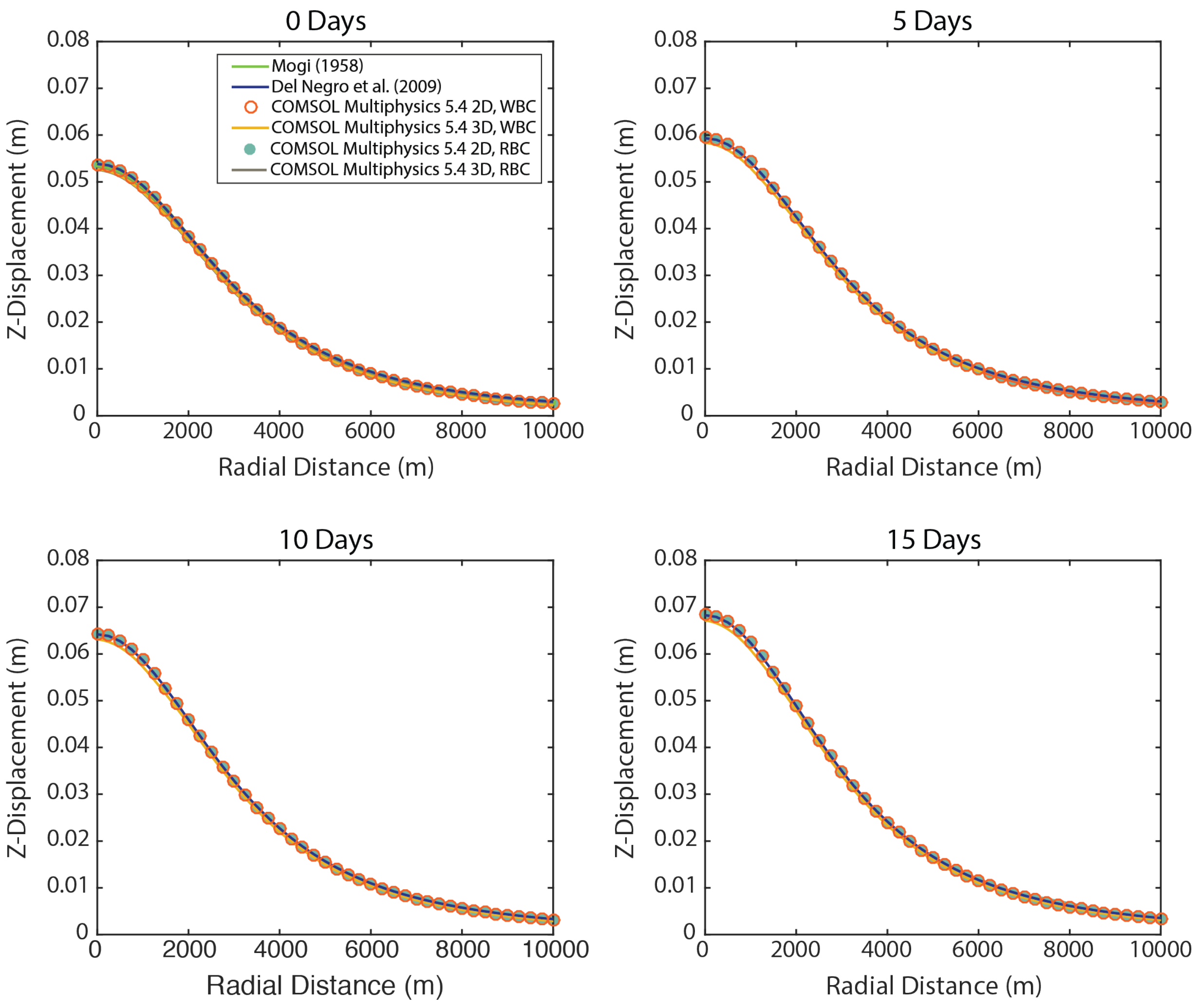

Figure S3: Figure showing the compatibility of the elastic analytical solution of Mogi (1958), the viscoelastic analytical solution of Del Negro (2009), the 2D COMSOL Multiphysics 5.4 FEMs (Gregg et al., 2012), and the 3D COMSOL Multiphysics 5.4 FEMs (Cabaniss et al., 2018). COMSOL Multiphysics 5.4 models built using a base Winkler boundary condition (WBC) are also benchmarked to those using a base roller boundary condition (RBC).

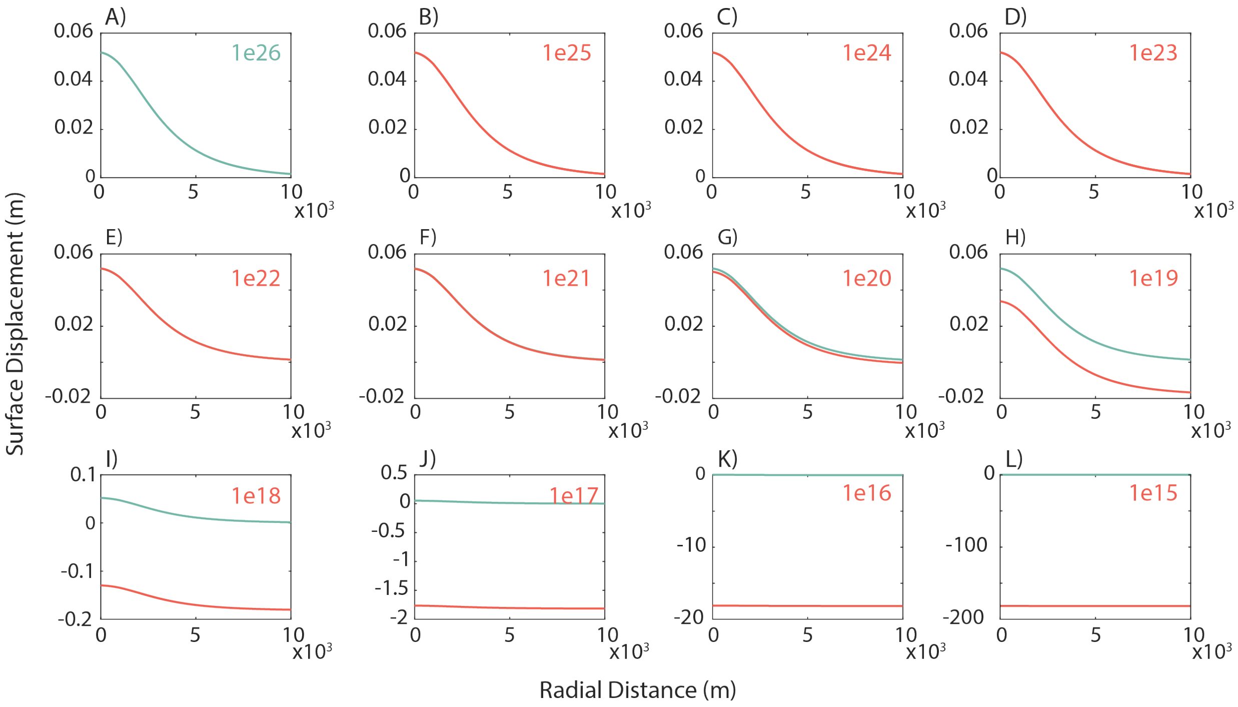

Figure S4: Results of the Winkler foundation and elastic roller boundary condition benchmark experiment show model-predicted surface displacement is in good agreement until the spring constant (red number) is weakened by ~6 orders of magnitude.

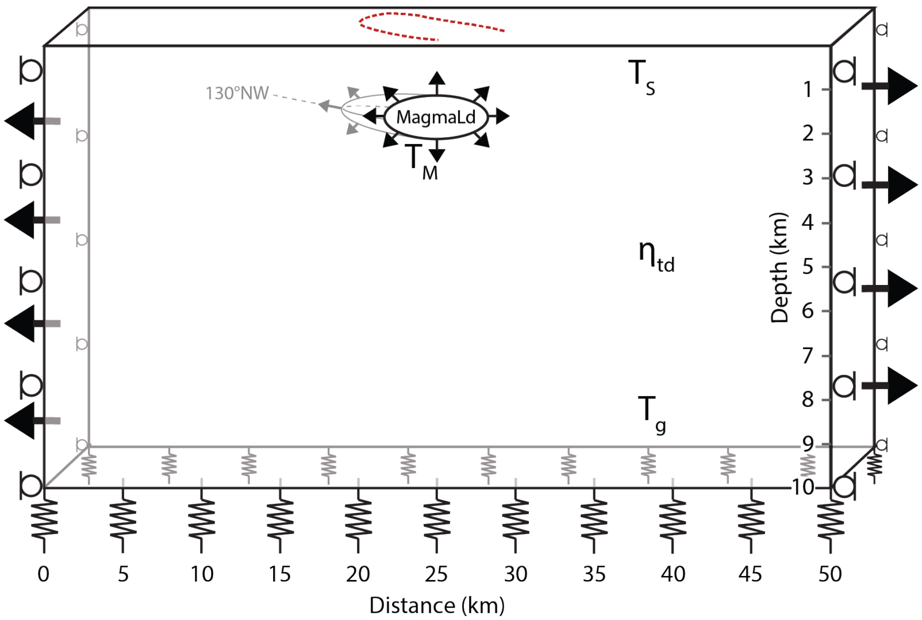

Figure S5. Three-dimensional model setup. An initial background thermal regime is calculated using a constant geothermal gradient, T_g, of 30 °C/km, where the surface temperature, T_S=0 °C and the temperature of the magma reservoir boundary, T_M, is held constant at 1200 °C. A steady-state thermal structure is used, and from this, the temperature-dependent viscosity, η_{td}, is calculated. As in Galgana et al. (2011) a Winkler boundary condition is applied to the bottom of the model to account for any flexural forces, and roller boundary conditions are applied to the lateral model-bounding surfaces. The left and right vertical surfaces have boundary conditions applied that allow a velocity to be prescribed to them (*pv*), simulating Juan de Fuca ridge extension of 60 mm/year. These surfaces are indicated by arrows, which show the direction of applied tectonic stress.

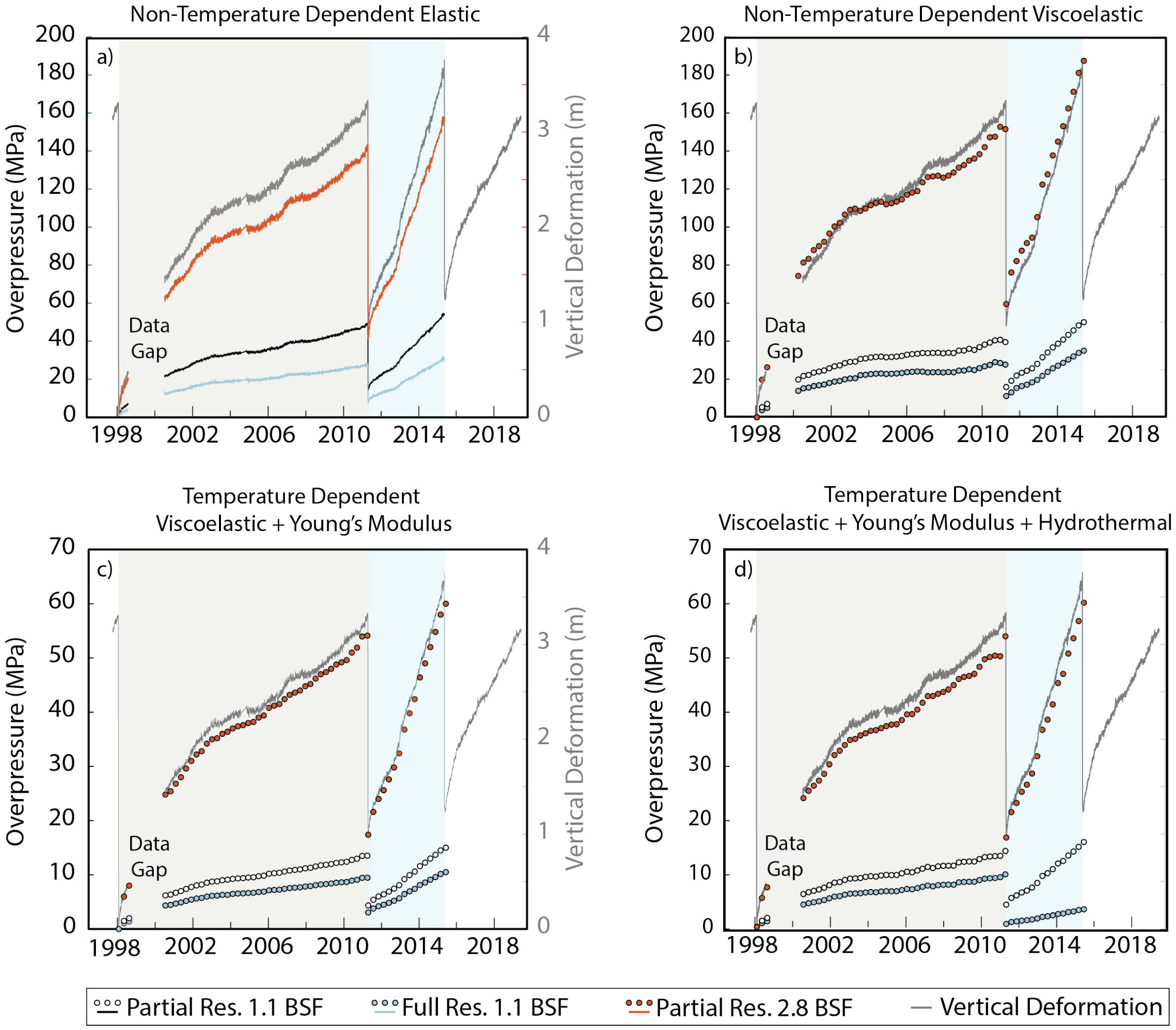

Figure S6. Reservoir overpressure conditions required to reproduce the observed deformation at the location of the Center BPR at Axial Seamount for a variety of reservoir geometries and for each tested rheology.
